# Involvement of GluR2 up-regulation in neuroprotection by electroacupuncture pretreatment via cannabinoid CB1 receptor in mice

**DOI:** 10.1038/srep09490

**Published:** 2015-04-01

**Authors:** Zhaoyu Liu, Xiyao Chen, Yang Gao, Sisi Sun, Lei Yang, Qianzi Yang, Fuhai Bai, Lize Xiong, Qiang Wang

**Affiliations:** 1 Department of Anesthesiology, Xijing Hospital, Fourth Military Medical University, Xi'an 710032, China; 2 Department of Physiology, Fourth Military Medical University, Xi'an 710032, China

## Abstract

We investigated whether glutamate receptor subunit 2 (GluR2) is involved in EA pretreatment-induced neuroprotection via cannabinoid CB1 receptors (CB1R) after global cerebral ischemia in mice. Two hours after electric acupuncture (EA) pretreatment, global cerebral ischemia (GCI) was induced by bilateral common carotid artery occlusion (BCCAO) for 20 min. The GluR2 expression was examined in the hippocampus after reperfusion. Cell survival, neuronal apoptosis, the Bax/Bcl-2 ratio and neurological scores were evaluated at 24 h after BCCAO in the presence or absence of the GluR2 inhibitor. Furthermore, the GluR2 was determined in the presence and absence of CB1R inhibitor. Our results showed EA pretreatment enhanced expression of GluR2 in the hippocampus 2 h after reperfusion. Moreover, EA pretreatment improved neurological outcome, promoted cell survival, inhibited neuronal apoptosis, and decreased the Bax/Bcl-2 ratio after reperfusion. GluR2 knockdown by GluR2 siRNA effectively reversed the beneficial effects of EA pretreatment. Furthermore, CB1R siRNA and two CB1R antagonists blocked the elevation of GluR2 expression by EA pretreatment, whereas the two CB1R agonists up-regulated GluR2 expression as EA pretreatment. In conclusion, GluR2 up-regulation is involved in neuroprotection of EA pretreatment against GCI through CB1R, suggesting that GluR2 may be a novel target for stroke intervention.

Stroke is a leading cause of death and disability^1^. Ischemic brain injury is the major pathophysiology for a stroke to have a poor outcome. Medical researchers wish to intervene and reduce this injury, but there are few effective pharmacological treatments once an ischemic stroke occurs. Thrombolytics, such as Activase and TNKase, have been a significant advance, but these must be administered within 3–4.5 h of ischemia's onset. This narrow therapeutic window limits thrombolytics' practical application in much of the world^2^. Thus, there is still significant unmet need for acute stroke treatments that are beyond help from thrombolytics.

Glutamate accumulation, which occurs immediately after ischemia, results in excessive stimulation of glutamate receptors and leads to neurotoxicity^3^. Glutamate activates two major subfamilies of ligand-gated postsynaptic receptors: a-Amino-3-hydroxy-5-methyl-4-isoxazolepropionic acid receptors (AMPAR) and N-methyl-D-aspartate receptor (NMDAR)^4^,^5^. Initial studies focused on NMDA-type glutamate receptors as a critical mediator in focal ischemic injury. Subsequent studies support a more central role for AMPA-type glutamate receptors in the selective pattern of neuronal loss in the hippocampus, which is associated with global ischemia^6^. Many neuroprotective drugs are designed to inhibit ischemia-induced excitotoxicity by acting as glutamate receptor antagonists. However, the clinical application of glutamate receptor antagonists is limited due to directly blocking receptor physiological function^7^,^8^. An ideal neuroprotective agent should be able to block glutamate-mediated neurotoxicity without impairing physiological glutamatergic neurotransmission.

AMPARs are heterogenic complexes composed of glutamate receptor subunit 1–4 (GluR1-GluR4). All subunits are permeable to both Na^+^ and Ca^2+^ ions with the exception of GluR2, which is uniquely impermeable to Ca^2+^. The GluR2 subunit dictates Ca^2+^ permeability of AMPA receptor channels^5^,^9^. GluR2's expression in neurons is not static and is altered after seizures, ischemic insults, antipsychotics, drugs abuse, and corticosteroids. Considerable evidence shows global ischemia triggers down-regulation of GluR2 protein abundance and enhances AMPAR-mediated Ca^2+^ influx in vulnerable CA1 pyramidal neurons before neuronal death begins^10^. A rise in intracellular Ca^2+^ may spur events leading to cell death, suggesting GluR2-lacking AMPAR-mediated excitotoxicity plays a critical role in cerebral ischemic insults^11^.

Previous studies showed anandamide directly inhibits AMPA receptor subunit recombinant in Xenopus oocytes, revealing the close relationship between endocannabinoids and glutamate receptors. Other research showed the endocannabinoid 2-arachidonylglycerol (2-AG) and anandamide (AEA) are activated in neuronal cells in response to excitotoxicity induced by AMPAR activation. Activating the endogenous cannabinoid system plays a neuroprotective role through the cannabinoid receptors. The inhibitor of endocannabinoid uptake, UCM707, protected specifically against AMPA-induced excitotoxicity by activating CB1R and CB2 receptor (CB2R)^12^. Excitotoxicity rapidly raised endocannabinoid levels in the hippocampus. These endocannabinoids induced protective mechanisms in wild-type mice conversely, but could not be triggered in CB1R knockout mice^13^,^14^. WIN55.212-2 inhibited the release of glutamate, and a CB1R antagonist SR141716A counteracted the role of WIN55.212-2^15^. Above referring findings suggest endogenous cannabinoid system activation can resist excitotoxicity induced by overly activating AMPAR. The effect has close relationship with CB1 cannabinoid receptors.

Acupuncture is critical to traditional Chinese medicine, while EA combines traditional Chinese acupuncture and modern electrical techniques. Our previous investigations demonstrated EA pretreatment at Baihui (GV 20) acupoint modulated the endocannabinoid system, in which it increased endocannabinoid ligands (2-AG and AEA) production and induced ischemic tolerance through action on cannabinoid receptors^16^,^17^. The link between the endocannabinoid system and GluR2 for EA pretreatment-induced neuroprotection is not fully understood. Based on these findings, we tested the hypothesis that GluR2 up-regulation is involved in EA pretreatment-induced neuroprotection against global cerebral ischemia via CB1R in mice.

## Results

### GluR2 expression was down-regulated in hippocampus at 2 h after reperfusion

**Figure 1:**
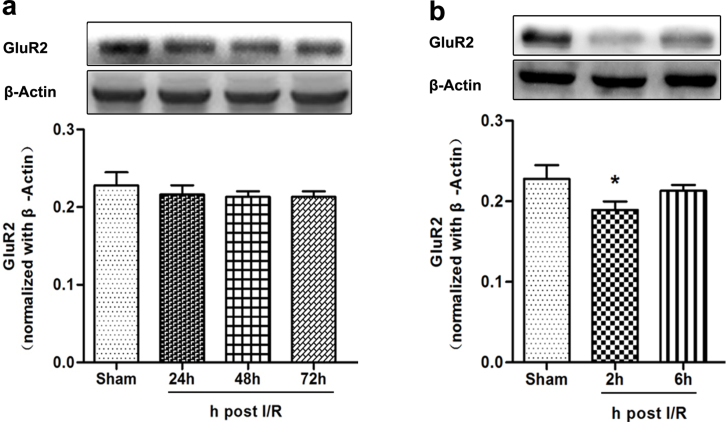
Expression of GluR2 protein in hippocampus after global cerebral ischemia (*n* = 5). (a) Western blot analysis of GluR2 protein expression in sham, global cerebral ischemia 24 h, global cerebral ischemia 48 h, and global cerebral ischemia 72 h groups. The upper part is the photograph of GluR2 and its corresponding β-Actin bands. The GluR2 protein expression level did not significantly change at 24 h, 48 h, and 72 h after reperfusion compared to the Sham group. (b) Western blot analysis of GluR2 protein expression in hippocampus in the sham, global cerebral ischemia 2 h, global cerebral ischemia 6 h. The upper part is the photograph of GluR2 and its corresponding β-Actin bands. (*P < 0.05 vs. Sham).

We examined insult-induced global ischemia alterations in GluR2 protein expression by western blot analysis. Band density analysis indicated global cerebral ischemia did not significantly alter GluR2 protein expression in the hippocampus as late as 72 h after reperfusion (Fig. 1a). However, a marked reduction in GluR2 subunit abundance in hippocampus was visible 2 h after reperfusion (*P* = 0.013; Fig. 1b).

### EA pretreatment up-regulated GluR2 in hippocampal CA1 pyramidal neurons after reperfusion

**Figure 2:**
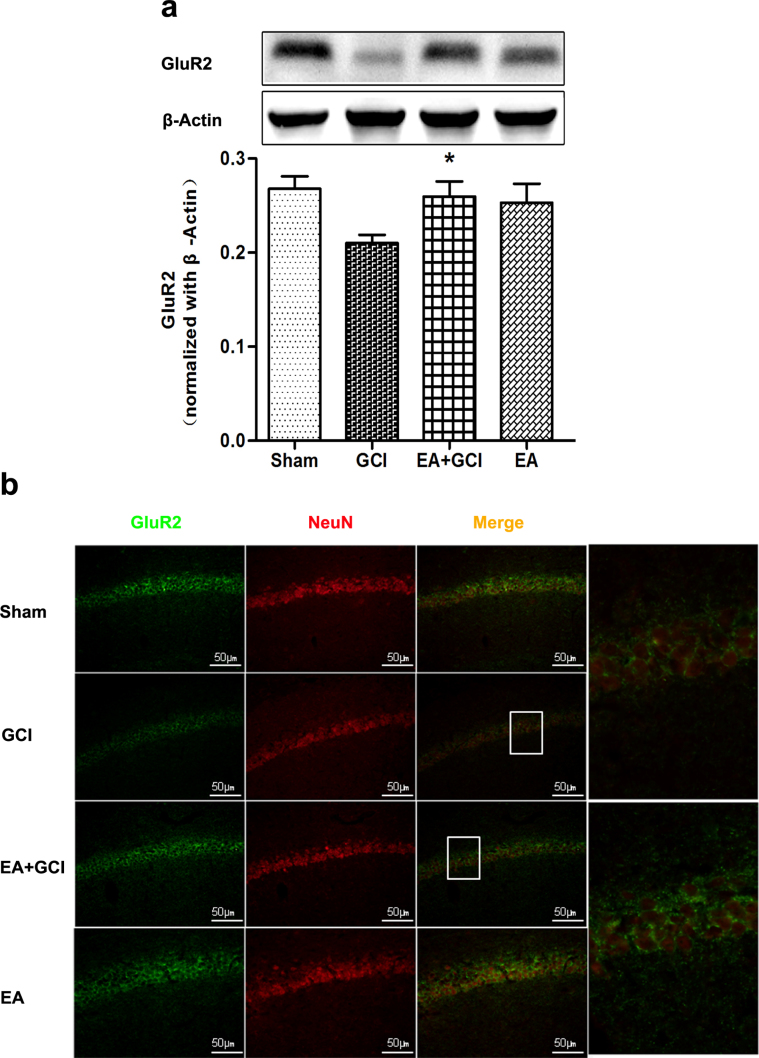
Effect of EA pretreatment on GluR2 expression in hippocampus after global cerebral ischemia *(n* = 5*)*. (a) Western blot analysis of the expression of GluR2 protein in the Sham group, GCI group, EA+GCI group, and EA group. The upper part is the photograph of GluR2 and its corresponding β-Actin bands. (*P < 0.05 vs. GCI). (b) Double immunofluorescent staining for GluR2 (in green) and neurons (in red) in the hippocampus 2 h after reperfusion. EA pretreatment increased cell-specific expression of GluR2 (Upper) immunolabeling in the CA1 pyramidal cell layer. Scale bars = 50 μm.

EA pretreatment 2 h prior to global cerebral ischemia resulted in GluR2 up-regulation according to western blotting, and double-labeled immunolabeling. The analysis indicated pretreatment with EA significantly increased GluR2 protain in the hippocampus compared to the GCI group (*P* = 0.020; Fig. 2a). Moreover, the analysis of double-labeled immunolabeling demonstrated the same result (*P* = 0.043; Fig. 2b). The level of GluR2 protein expression, the number of GluR2-positive cells co-localized with NeuN positive neurons showed no difference between the EA+GCI and Sham groups.

### GluR2 knockdown abolished the improvement of neurological score by EA pretreatment after reperfusion

**Figure 3:**
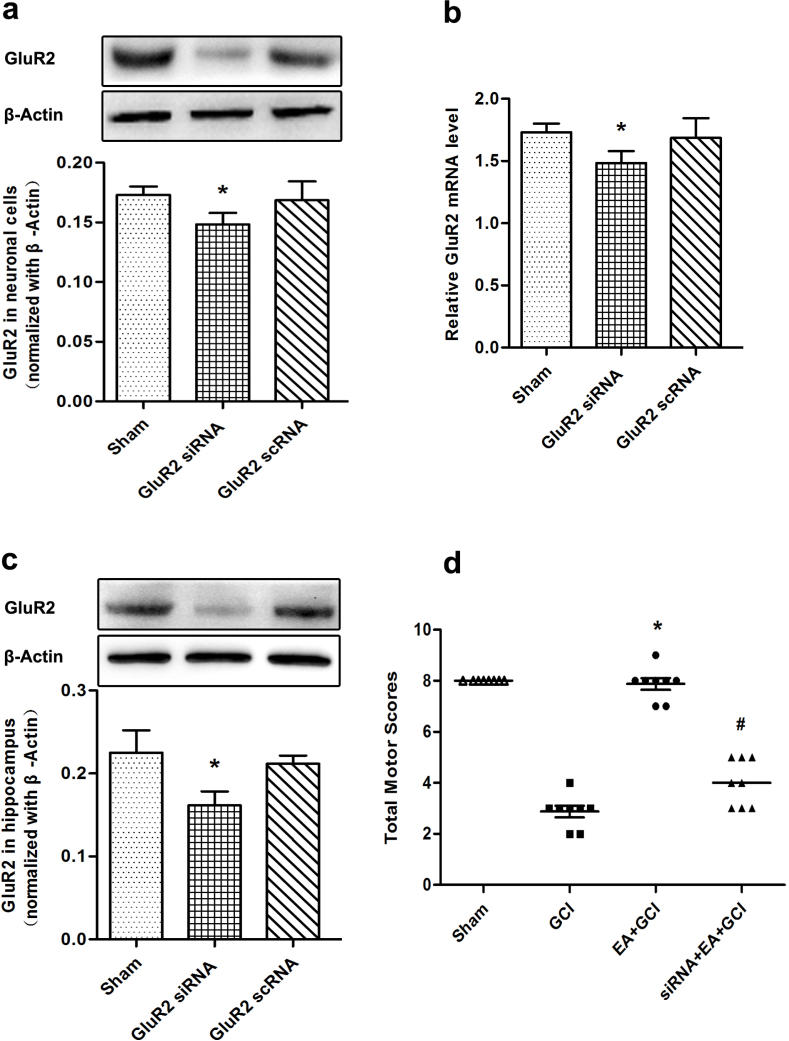
Neurological scores at 24 h after reperfusion in the mice with 20 min of global cerebral ischemia (*n* = 8). (a, c) Western blotting analysis of GluR2 at 72 h after cell transfection and hippocampal injection of GluR2 siRNA Mm-Gria2-2. Mm-Gria2-2 remarkably reduced the expression of GluR2 protein in neuronal cells and hippocampus(*P < 0.05 vs. Sham). The upper part is the photograph of GluR2 and its corresponding β-Actin bands. (b) Analysis of the GluR2 mRNA in the neuronal cells. GluR2 siRNA remarkably decreased the mRNA of GluR2 (*P < 0.05 vs. Sham). (d) Neurological scores 24 h after reperfusion in the mice with 20 min of global cerebral ischemia. Pretreatment with EA significantly improved the neurological scores, whereas the GluR2 siRNA reversed the beneficial effect of EA pretreatment (*P < 0.05 vs. GCI; ^#^*P* < 0.05 vs. EA+GCI).

We selected a GluR2 siRNA named Mm-Gria2-2 to knockdown GluR2 protein expression in neuronal cells and mice. Then investigate its efficacy by western blotting analysis and RT-PCR. A marked reduction in GluR2 expression was visible in the hippocampus and in neuronal cells (*P* = 0.034 and 0.025; Fig. 3a and 3c). At the same time, this siRNA decrease the mRNA of GluR2 in the cell experiment (*P* = 0.019; Fig. 3b). In the siRNA+EA+GCI group, the neurological scores were lower than in the EA+GCI group (*P* = 0.009; Fig. 3d) and were similar to those of the GCI group.

### GluR2 knockdown prevented the promotion of cell survival by EA and attenuated the inhibitory effect of EA pretreatment on cellular apoptosis after reperfusion

**Figure 4:**
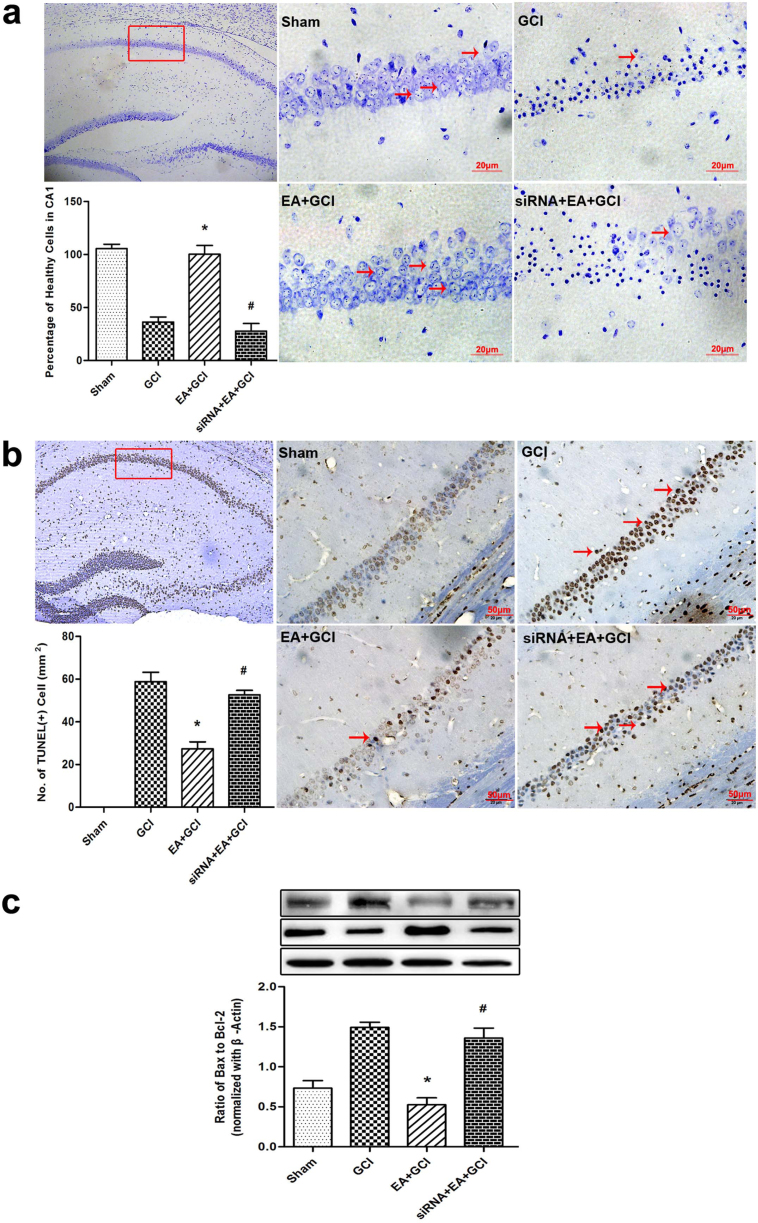
Neuronal survival, Neuronal apoptosis at 24 h after reperfusion in the mice with 20 min of global cerebral ischemia (*n* = 5). (a)Identification of neuronal survival by Nissl-staining in rat hippocampus 24 h after reperfusion. The cell counting showed the number of viable cells in the EA+GCI group is higher compared to the GCI group (*P < 0.05 vs. GCI). GluR2 siRNA decreased the number of viable cell compared to the EA+GCI group (^#^*P* < 0.05 vs. EA+GCI). Scale bars = 20 μm. (b) Neuronal apoptosis was assessed by TUNEL staining 24 h after reperfusion in the mice. The cell counting showed an attenuation in TUNEL-positive neuronal in the hippocampus in the EA+GCI group compared to the GCI group (*P < 0.05 vs. GCI). In siRNA-treated mice, TUNEL- positive cells were significantly higher than in EA-treated mice (^#^*P* < 0.05 vs. EA+GCI). Scale bars = 50 μm. (c) The ratio of Bax/Bcl-2 expression in ischemic mice pretreated with EA or GluR2 siRNA before global cerebral ischemia was analyzed by western blot. The upper part is the photograph of Bax or Bcl-2 and its corresponding β-Actin bands. (*P < 0.05 vs. GCI; ^#^*P* < 0.05 vs. EA+GCI).

At 24 h after reperfusion, the mice were decapitated and underwent Nissl staining. Pretreatment with EA significantly improved the percentage of viable neurons in the hippocampus compared to that of the GCI group. The GluR2 siRNA given 72 h before the onset of each EA pretreatment prevented the beneficial effect of EA pretreatment (*P* = 0.013; Fig. 4a). There were no statistical differences in the percentage of viable neurons between the GCI and siRNA+EA+GCI groups.

Neuronal cell death was evaluated 24 h after reperfusion in each group. Morphologically damaged neurons were positive for TUNEL staining. No positive TUNEL staining (brown) was detected in the Sham group brain sections. However, a large number of TUNEL-positive cells in the hippocampus were seen in the GCI and siRNA+EA+GCI groups. By contrast, only a small amount of TUNEL-positive cells in the EA+GCI group were observed (*P* = 0.040; Fig. 4b).

Bcl-2 and Bax proteins play important roles in apoptosis. We examined the expression of Bcl-2 and Bax in the hippocampus tissue 24 h after reperfusion. A marked reduction in Bax protein abundance in the hippocampus was visible by EA pretreatment. The Bax protein expression in the siRNA+EA+GCI group was significantly higher than in the EA+GCI group. The Bcl-2 protein expression in the EA+GCI group was higher than those of GCI group, and a reduction in Bcl-2 protein abundance was obvious in the siRNA+EA+GCI group. Our results showed a significant difference in Bax/Bcl-2 ratio in the EA group and the siRNA+EA+GCI group (*P* = 0.035; Fig. 4c).

### Pretreatment with 2-AG up-regulated GluR2 through CB1R, but not CB2R

**Figure 5:**
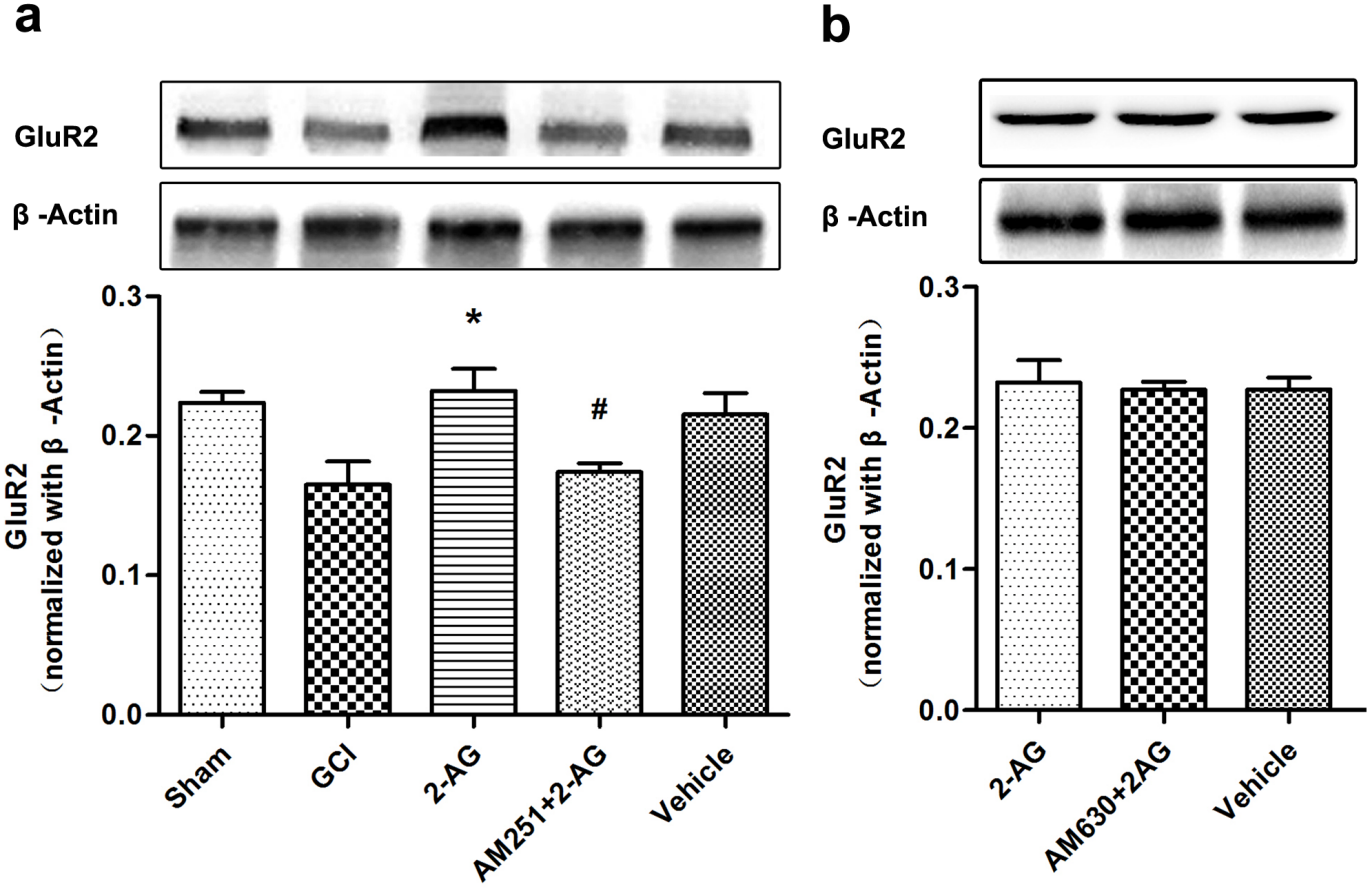
Pretreatment with 2-AG up-regulated GluR2 expression via CB1R (*n* = 5). (a) Western blot analysis of GluR2 expression in Sham, GCI, 2-AG, AM251+2-AG and vehicle groups (*P < 0.05 vs. GCI; *^#^P* < 0.05 vs. 2-AG). The upper part is the photograph of GluR2 and its corresponding β-Actin bands. (b) Western blot analysis of GluR2 expression in 2-AG, AM630+2-AG and vehicle groups. The upper part is the photograph of GluR2 and its corresponding β-Actin bands.

The effect of pretreatment with endocannabinoid 2-AG on GluR2 expression was investigated by western blotting. 2-AG administration 30 min prior to global cerebral ischemia resulted in up-regulation of GluR2 expression in the hippocampus. AM251, a CB1R selected specific antagonist, was injected 30 min before 2-AG administration and reversed the regulatory effect of 2-AG on GluR2 expression (*P* = 0.026; Fig. 5a). However, AM630, a CB2R selected specific antagonist, had no influence on the GluR2 expression compared with the 2-AG group (Fig. 5b). There were no statistical differences in the GluR2 expression level between the 2-AG and Vehicle groups.

### Pretreatment with CB1R agonists up-regulated GluR2 expression

**Figure 6:**
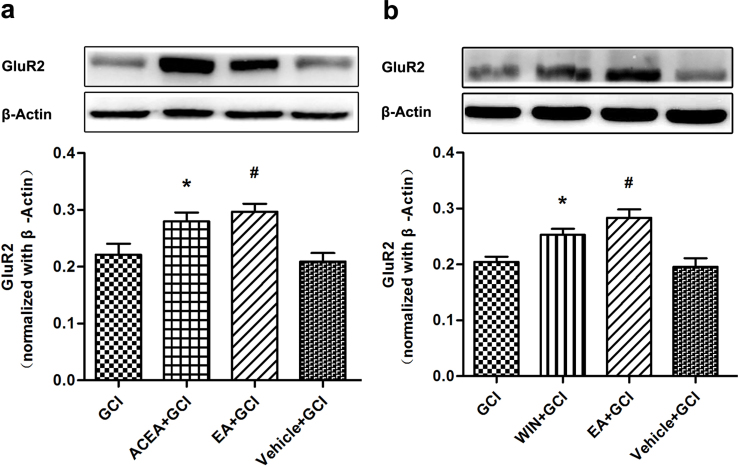
Pretreatment with CB1R agonists up-regulated GluR2 expression (*n* = 5). (a, b) Western blot analysis of GluR2 expression in GCI, ACEA+GCI, WIN55212-2+GCI, EA+GCI and Vehicle+GCI group. The upper part is the photograph of GluR2 and its corresponding β-Actin bands. (*P < 0.05 vs. GCI; ^#^*P* < 0.05 vs. GCI).

Two CB1R agonists, ACEA and WIN55212-2, were administered intraperitoneally 30 min before global cerebral ischemia. We found that 2 h after reperfusion, the expression of GluR2 in the ACEA+GCI and WIN55212-2+GCI groups were higher than in the GCI and Vehicle +GCI groups (*P* = 0.001 and 0.015; Fig. 6a and 6b).

### CB1R antagonists and CB1R siRNA dampened the regulatory effect of EA pretreatment on GluR2 expression

**Figure 7:**
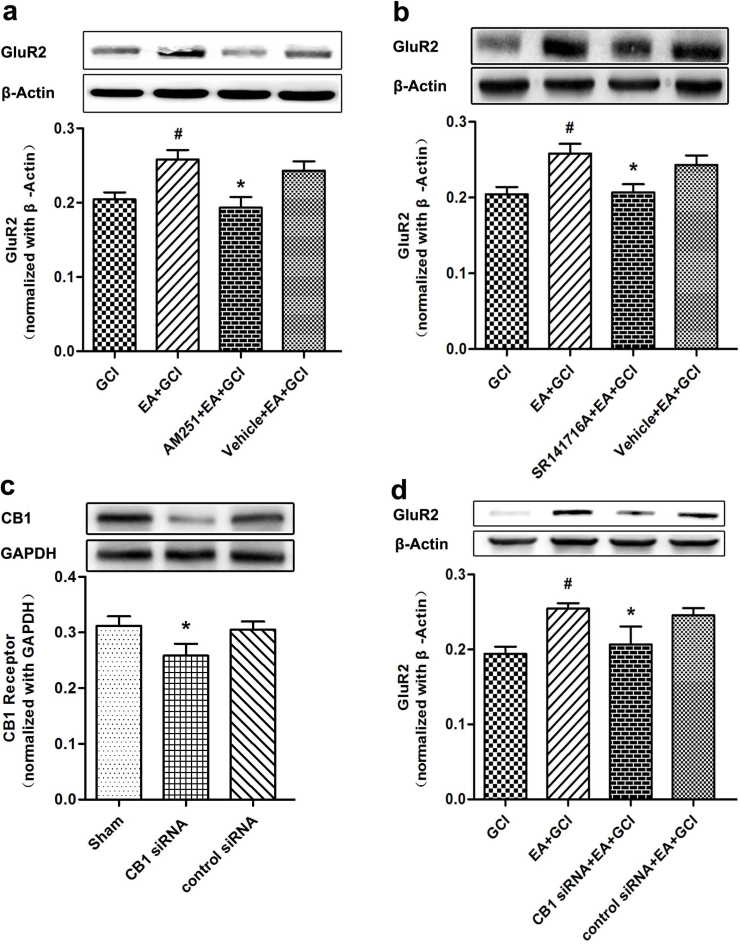
CB1R antagonists and CB1R siRNA dampened the regulatory effect of EA pretreatment on GluR2 expression (*n* = 5). (a, b) Western blot analysis of GluR2 expression in GCI, EA+GCI, AM251+EA+GCI, SR141716A+EA+GCI and Vehicle+EA+GCI. The upper part is the photograph of GluR2 and its corresponding β-Actin bands. (*P < 0.05 vs. EA+GCI; ^#^*P* < 0.05 vs. GCI). (c) Western blotting analysis of CB1R expression at 72 h after lateral ventricle injection of CB1R siRNA. CB1R siRNA remarkably reduced the expression of CB1R protein (*P < 0.05 vs. Sham). The upper part is the photograph of CB1R and its corresponding GAPDH bands. (d) Western blot analysis of GluR2 expression in GCI, EA+GCI, CB1 siRNA+EA+GCI, control siRNA+EA+GCI. There was a significant difference between EA+GCI group and CB1 siRNA+EA+GCI group. (*P < 0.05 vs. EA+GCI; ^#^*P* < 0.05 vs. GCI).

CB1R siRNA and two CB1R antagonists, AM251 and SR141716A, were used to test whether GluR2 up-regulation by EA pretreatment was CB1R dependent. The GluR2 expression in the AM251+EA+GCI group was significantly lower compared to EA+GCI and Vehicle+EA+GCI (*P* = 0.045; Fig. 7a). We found no significant difference between the GCI group and the AM251+EA+GCI group. We used SR141716A, another CB1R antagonist injected intracerebrally, to interfere with CB1R. Its effect was similar to AM251 (*P* = 0.033; Fig. 7b). To get solid evidence, we used CB1R siRNA injected intracerebrally to interfere with CB1R. Western blotting analysis proved the CB1R siRNA resulted to effective CB1R knockdown (*P* = 0.043; Fig. 7c). The expression of GluR2 in the CB1R siRNA+EA+GCI group was significantly decreased compared with that in the EA+GCI and control siRNA+EA+GCI groups (*P* = 0.048; Fig. 7d).

## Discussion

The promising approach to reduce ischemic brain injury involves enhancing endogenous protective mechanisms^18^. Preconditioning, a prior exposure of tissues or organs to a stimulus or drug, is one such approach to reduce ischemia- or severe hypoxia-induced injury to the tissues or organs^19^. A potent way to trigger the endogenous protective responses, preconditioning activates several endogenous signaling pathways and result in protection against ischemia^20^. Therefore, further research is necessary to define the precise molecular crosstalk and to optimize a novel way of handling cerebral ischemia.

Transient forebrain or global cerebral ischemia as a consequence of cardiac arrest, cardiac surgery, or near drowning induced a selective pattern of neuronal loss in the brain^21^. The most vulnerable neurons, in terms of insult duration, are the hippocampal CA1 pyramidal cells. That selective neuronal injury is Ca^2+^ dependent and likely reflects the activation of Ca^2+^-permeable AMPA receptor channels^22^. The GluR2 subunit governs the permeability of AMPAR to Ca^2+^ 9.

The direct evidence demonstrated that excessive stimulation of AMPA-induced exitotoxicity rapidly raised hippocampal levels of anandamide and induced protective mechanisms in principal hippocampal neurons of wild-type mice. Conversely, these protective mechanisms could not be triggered in mutant mice that lacked CB1R expression in principal hippocampal neurons^23^.

Our previous study demonstrated that, similar to the ischemic tolerance induced by ischemic preconditioning, EA pretreatment at Baihui acupoint (GV 20) before ischemic insult could produce evident neuroprotection. Additional work demonstrated a series of molecules were involved in the neuroprotection of EA pretreatment^24^,^25^. One of the most compelling findings was that EA pretreatment-induced neuroprotection was possibly mediated through an endocannabinoid-related mechanism. In this system, EA pretreatment increased production of endocannabinoid 2-AG and AEA, and it elicited protective effects against transient cerebral ischemic injury via CB1R or CB2R^26^. However, the manner by which such molecules and receptors networked, and their putative role, remained undetermined. More evidence is needed for EA pretreatment to be accepted clinically.

In this study, we postulated the link between EA pretreatment and regulation of GluR2. First we explored GluR2 expression changes after global cerebral ischemia. In former research, Mongolian gerbils underwent temporary 5 min bilateral occlusion of the carotid arteries to execute global ischemia model. This led to a marked reduction in the level of GluR2 protein in CA1 at 72 h^27^,^28^. In another study, Sprague-Dawley rats underwent global ischemia by four-vessel occlusion for 10 min. This model reduced GluR2 subunit abundance in CA1, evident at 48 h^29^. In contrast to those researches, we chose a BCCAO model to induce transient global cerebral ischemia in C57BL/6 mice according to previous reports^30^,^31^. We found global cerebral ischemia down-regulated the expression of AMPAR subunit GluR2 in hippocampus as early as 2 h after reperfusion.

To investigate whether GluR2 was involved in EA pretreatment-induced neuroprotection, we assessed effect of EA pretreatment on the expression of GluR2 protein in the hippocampus. Our results showed EA pretreatment significantly up-regulated GluR2 expression after reperfusion in comparison with the GCI group. This implied that GluR2 up-regulation contributed to developing EA-induced neuroprotection against severe ischemic insults. These results were consistent with previous studies in which GluR2 up-regulation specifically mediated protective signaling at the early stage of ischemia^32^. We further examined whether inhibition of GluR2 altered the EA pretreatment's effect on outcomes after ischemic insults. Knockdown of GluR2 expression by GluR2 siRNA reversed EA pretreatment neuroprotection, in which EA improved cell survival and neurobehavior function after reperfusion. These results indicated that up-regulation of GluR2 following ischemia attack may act as an essential step to switch the cells to a protective state against ischemia insults. Failure of such up-regulation results in loss of neuroprotection, as we observed in the presence of GluR2 siRNA.

Strong evidence showed that during the cerebral ischemia, a lot of Ca^2+^ flowed into cell directly through GluR2-lacking AMPAR. A rise in intracellular calcium Ca^2+^ is thought to lead to cell death, and apoptosis plays an important role in the brain tissue after transient cerebral ischemia^6^,^33^. Bax collaborates with Bak to permeabilize the outer membrane of mitochondria, which leads to apoptotic cascade when the cell suffers from injuries. By contrast, the Bcl-2 family proteins inhibit this process. The balance between Bax and Bcl-2 is critical to turning on or off the cellular apoptotic machinery^34^. Previous studied have verified that an increase of the neuroprotective factor GluR2, together with the anti-apoptotic Bcl-2, appeared to be linked with reduced argyrophilic signals in the arousal hibernating states^35^. Moreover, vascular endothelial growth factor (VEGF) exerted its anti-excitotoxic effects on motor neurons through up-regulation of GluR2 subunit of AMPA receptors and the anti-apoptotic molecule Bcl-2^36^. In the present study, the knockdown of GluR2 expression clearly reversed the beneficial effect in which EA pretreatment significantly reduced apoptotic cell numbers and the Bax/Bcl-2 expression ratio in the hippocampus. The data further supports that the neuroprotective effect of EA pretreatment against ischemia-induced apoptosis might be at least partly mediated by regulating Bax and Bcl-2 expression through GluR2 up-regulation.

It was unclear if GluR2 up-regulation mechanisms induced by EA pretreatment may be associated with upstream modulator or mediators. In the central nervous system, endocannabinoids have multiple functions, which occur mainly via highly localized stimulation of CB1R in the basal ganglia, hippocampus, and cortex. Our previous study showed EA pretreatment increased the production of endocannabinoid 2-AG and AEA, and up-regulated the CB1R neuronal expression of in rat brains. EA pretreatment-induced neuroprotective effects were attenuated by AM251 or CB1R knockdown. Those findings indicated that EA pretreatment elicited protective effects against transient cerebral ischemia through CB1R^37^,^38^,^39^. In the present study, AM251, a selective antagonist that blocks 2-AG from binding the CB1R, inhibited EA pretreatment-induced GluR2 up-regulation and neuroprotective effects. AM630 (a selective CB2R antagonist) had no effect on the up-regulation of GluR2, indicating that the up-regulation of GluR2 involved in EA pretreatment-mediated neuroprotection is dependent on CB1R. Furthermore, administration of the two CB1R agonists ACEA and WIN55212 before global cerebral ischemia up-regulated GluR2 expression, which mimicked the effect of EA pretreatment. Conversely, administering CB1R selective antagonist AM251 and SR141716A abolished the up-regulation of GluR2 induced by EA pretreatment. For more solid evidence, we used CB1R siRNA to interfere with CB1R, and found the CB1R knockdown conferred the same result as AM251 and SR141716A. These findings indicated that the up-regulation of GluR2 involved in EA pretreatment-mediated neuronal protection is dependent on CB1R.

In conclusion, our previous work and current results strongly suggest that GluR2 upregulation-mediated anti-apoptosis was involved in EA pretreatment through the CB1 receptor. Further investigation should try to elucidate the detailed signal cascades underlined in the CB1R–GluR2 pathway of the EA pretreatment. However, present findings may represent a novel profile for EA pretreatment-induced neuroprotection mechanisms against global cerebral ischemia in mice. The results also pave the way for other GluR2-related interventions to protect against neuronal cell death due to stroke and other excitotoxic neuronal disorders.

## Methods

### Animals

All efforts were made to minimize the number of animals used, minimize suffering, comply with protocol approved by the Ethics Committee for Animal Experimentation, and proceed according to the Guidelines for Animal Experimentation of the Fourth Military Medical University. Male C57BL/6 mice, aged 12 weeks and weighing 18 to 22 g, were provided by the Experimental Animal Center of the Fourth Military Medical University and housed under controlled conditions: a 12 hours light/dark cycle, temperatures at 21 ± 2°C, and humidity at 60–70% for at least 1 week prior to drug treatment or surgery. The mice freely accessed a standard rodent diet and tap water.

### EA pretreatment

We performed EA pretreatment as described in our previous studies^40^,^41^. After 12 h of fasting, the mice were anesthetized with 10% chloral hydrate intraperitoneal injection (300 mg/kg) and inhaled oxygen by face mask at a flow rate of 1 L/min. The “Baihui” acupoint (GV 20), located at the intersection of the sagittal midline and the line linking the two ears, was stimulated with the intensity of 1 mA and frequency of 2/15 Hz for 30 min using the Hwato electronic acupuncture treatment instrument (model No. SDZ-V, Suzhou Medical Appliances Co., Ltd, Suzhou, China). The core temperature of the mice was maintained at 37.0 ± 0.5°C during EA pretreatment by surface heating or cooling. The right femoral artery was cannulated for continuous blood pressure monitoring and arterial blood sampling. Arterial blood gases and plasma glucose were measured at the onset of EA, 15 min after EA, and at the end of EA. pO2, pCO2, and pH were quantified using a blood gas analyzer (ABL700, Radiometer, Denmark), and plasma glucose was measured with a blood glucose meter (OneTouch UltraEasy, Johnson & Johnson, USA).

### Global cerebral ischemia and regional cerebral blood flow

The mice freely accessed water but fasted for 12 h before surgery. This study used bilateral common carotid artery occlusion (BCCAO) as a global cerebral ischemia (GCI) model^42^. Briefly, mice were anesthetized with 10% chloral hydrate intraperitoneal injection (300 mg/kg). A ventral midline incision was made to expose the trachea. The common carotid arteries were carefully separated from the surrounding tissues and vagus nerve and exposed. Then, both common carotid arteries were ligated with 5-0 silk sutures. The laser Doppler flowmeter (PeriFlux System 5000; Perimed, Stock-holm, Sweden) was used to measure regional cerebral blood flow (rCBF) (2–3 mm lateral to the bregma) from anesthetic induction to 5 min after reperfusion^43^. Only the mice whose mean cortical CBF should be reduced to <20% of the preischemic value were used for data analysis. After 20 min of cerebral ischemia, the ligatures were removed to allow blood reflow through the carotid arteries. The incision was sutured using 4-0 Mersilk (Ethicon, Johnson & Johnson, Somerville, NJ). During the surgical procedure, the pericranial temperature was monitored using a temperature probe and maintained at 37.0 to 37.5°C with a heating pad. After surgery, animals were placed in a warm surrounding (30–33°C) to avoid biased results from hypothermia.

### Transfection of GluR2 siRNA into the mouse brain

C57BL/6 mice were treated with GluR2 siRNA Mm-Gria2-2 (QIAGEN, Germany) and control siRNA (QIAGEN, Germany). Under anesthesia with 10% chloral hydrate (300 mg/kg), a stainless steel cannula was stereotaxically implanted in the hippocampus. The stereotaxic coordinates were 2.06 mm posterior, 1.25 mm lateral to the bregma, and 1.5 mm below the skull's surface^44^. Mm-Gria2-2 and control siRNA dissolved in RNase-free water. 0.2 μL of the diluted mixture was stereotaxically delivered into the hippocampus. After recovering from anesthesia, the mice returned to their cages and had ad libitum access to food and water. The protein expression of GluR2 was evaluated 72 h post-transfection using Western blot.

### Transfection of GluR2 siRNA into neurons

To allow incorporation of siRNAs into neurons, 4-day-old neuronal cell line (HT22) were incubated in Dulbecco's medium without serum for 24 hr on the day before transfection. Transfection solution contained 2 μl of Oligofectamine, 40 μl of Opti-MEMI, and 2.5 μl of siRNA and was added to the neurons for 8 hr. Neurons treated in this manner are indicated as siRNA(+). Another group of transfection solution had been treated with scRNA in a similar manner. After this treatment, 87.5 μl of DMEM with 37.5 μl of serum was added to the neurons. The expression of mRNA of GluR2 was measured by RT-PCR 3 days after transfection.

### Drug administration

2-AG, a kind of endocannabinoid ligand, was blended with dimethyl sulfoxid (DMSO) and Tween-80, diluted with saline (DMSO: Tween-80: saline = 1:1:18), and delivered intraperitoneally 30 min before GCI at a dose of 2.5 mg/kg. AM251 (Tocris Bioscience, Britain) and SR141716A, two CB1R antagonists, were dissolved in 5% DSMO before use. The doses for AM251 and SR141716A (1 mg/kg) were chosen based on previous studies^45^ and were given 30 min before the next processing. Arachidony-2-chloroethylamide (ACEA), a selective CB1R agonist (Sigma, USA), and WIN55212-2, another CB1R agonist, were dissolved in 5% DSMO. They were delivered intraperitoneally 30 min before GCI at a dose of 2.5 mg/kg^46^. Mice were transfected with CB1R siRNA (Santa Cruz Biotecnology, sc-270168, USA) or control siRNA (Santa Cruz Biotecnology, sc-370007, USA) in accordance with our previous study^17^.

### Western blot analysis

Mice were decapitated under deep anesthesia, and the hippocampus was rapidly separated and frozen at −80°C until use. A membrane protein extraction kit (Beyotime, Nantong, China) homogenized frozen tissues. Each sample's protein concentration was estimated using a protein assay kit. Samples (30 μg) were loaded onto polyacrylamide-SDS gels. The gels were electrophoresed and then transferred to PVDF membranes. After that, the membranes were blocked with a blocking buffer using bovine serum albumin (Sigma, 3%) for 60 min and probed with primary antibodies overnight at 4°C. They were then incubated for one hour at room temperature with appropriate secondary goat-anti-rabbit antibodies (Beyotime, Nantong, China; 1:20000 dilution). This study used the following primary antibodies: anti-GluR2 rabbit poly-clonal antibody (1:200 dilution), rabbit anti-Bcl-2 antibody (Cell Signaling Technology, USA; 1:1000 dilution) and rabbit anti-Bax antibody (Epitomics, USA; 1:500 dilutions). Appropriate secondary horseradish peroxidase-conjugated goat-anti-rabbit antibodies (Pierce Biotechnology Inc.; 1:5000 dilution) were also used. The different protein forms were distinguished by molecular weight. Each sample had immune-blotting 3 times, and the final optical density value (relative to that of the internal standard) represents the average of these 3 separate analyses. Image analysis was accomplished with computerized analysis software assistance (Bio-Rad Laboratories, Hercules, CA).

### Immunofluorescent double staining

We performed double immunofluorescent staining to evaluate GluR2 co-localization and neuron-specific nuclear protein (NeuN). The sections fixed by 4% PFA were washed 3 times with PBS. The slides were simultaneously incubated with the primary antibodies anti-GluR2 (Abcam, USA; 1:200 dilution) and anti-NeuN (Millipore, USA; 1:2000 dilution). For double labeling, the primary antibodies were detected with FITC-conjugated secondary antibody (1:200; Jackson ImmunoResearch Laboratories) and Cy3-conjugated secondary antibody (1:200; Jackson ImmunoResearch, West Grove, PA, USA). The sections were examined under a fluorescence microscope (Olympus, Japan).

### GluR2 RNA extraction and quantitative RT–PCR

GluR2 total RNA for neuronal cells was extracted by TRizol reagent (Invitrogen, CA, USA). Quantitative mRNA expression analysis of target genes was performed with a real-time RT-PCR system. Reverse transcription PCR was performed using the PrimeScript RT reagent kit (TaKaRa, Dalian, China) following the manufacturer's instruction. Quantitative real-time PCR was performed using SYBR Premix Ex Taq II (TaKaRa, Dalian, China)). Expression of β-Actin was used as the internal control. The PCR primers for GluR2 and β-Actin were as follows: GluR2-forward CTATTTCCAAGGGGCGCTGAT, GluR2-reverse CAGTCCAGGATTACACGCCG; β-Actin-forward GCTCCTCCTGAGCGCAAG, β-Actin- reverse CATCTGCTGGAAGGTGGACA.

### Nissl Staining

Nissl staining detected surviving neurons 24 h after reperfusion. For Nissl staining, the paraffin slices were stained with 0.5% Nissl dye solution at 37°C for 25 min until they reached the desired depth of staining. After being rinsed in distilled water and dehydrated in graded serried of ethanol, the sections were immersed in xylene, mounted in neutral balsam, and then cover slipped. Only the neurons with Nissl's Body and the intact morphology were counted as surviving. Cell counting was performed on five randomly selected non-overlapping fields in the CA1 region of the hippocampus per slide. The survival index was defined thusly: surviving index (%) = 100× (number of surviving neurons/total number of neurons)^47^.

### TUNEL Staining

At 24 h after reperfusion, neuronal nuclear damage after ischemia was assessed in situ by terminal deoxynucleotidyl transferase-mediated 2'-Deoxyuridine 5'-Triphosphate-biotin nick end labeling (TUNEL) staining. The protocol for detecting DNA fragmentation was based on the method by with slight modification for use in paraffin sections. Briefly, TUNEL-positive neurons in hippocampus were counted in six random areas of sections per high-power field (×400), and represented as the percentage of 100 neurons as the cell apoptosis index.

### Neurological Tests

A recovery interval lasted 24 h after surgery. Mice then underwent a modified neurological examination designed to detect motor deficits. Briefly, mice were placed on a 10–20 cm screen (grid size 0.2 × 0.2 cm) that could be rotated from 0° (horizontal) to 90° (vertical). The mouse was placed on the horizontal screen, and the screen was then rotated into the vertical plane. The length of time the mouse held on to the vertical screen was recorded for a 15 second maximum (allowing a total of 3 points). Next, the mouse was placed at the center of a horizontal wooden rod (1.5 cm in diameter), and the length of time the mouse remained balanced on the rod was recorded for a 30 second maximum (allowing a total of 3 points). Finally, a prehensile traction test was administered. The length of time the mouse clung to a horizontal rope was recorded for a 5 second maximum. A total motor score (TMS) with 9 points possible was computed from these tests. The neurological tests were determined at 24 h, 48 h, and 72 h after reperfusion by an observer unaware of the grouping. The TMS we applied in the current study proved a more accurate method to evaluate global cerebral ischemia in mice^48^.

### Statistical analysis

SPSS 17.0 for Windows was used to perform all statistical analyses. All data, except for neurological deficit scores, are presented as mean ± S.E.M. and were analyzed by Student's t-test or a one-way analysis of variance. Between-group differences were detected with the 2-way ANOVA. Neurological scores were expressed as the median (range) and were analyzed with the Kruskal-Wallis test followed by the Mann-Whitney U-test for multiple comparisons. Values of *P* < 0.05 were considered statistically significant.

## Author Contributions

Z.L. and X.C. performed the experiment. Y.G., S.S. and Q.Y. analyzed the data. L.Y. and F.B. contributed to reagents/materials/analysis tools. Q.W. and L.X. designed the experiments and reviewed/edited the manuscript extensively. Z.L. wrote the manuscript and had full access to all the data in the study and takes responsibility for the integrity of the data and the accuracy of the data analysis.
